# Management of risperidone-induced hyperprolactinemia in children: a case report

**DOI:** 10.1192/j.eurpsy.2023.1080

**Published:** 2023-07-19

**Authors:** H. Belhadga, Z. Elmaataoui, H. Kisra

**Affiliations:** Hôpital psychiatrique universitaire arrazi de sale, Hôpital psychiatrique universitaire arrazi de sale, Sale, Morocco

## Abstract

**Introduction:**

Antipsychotics have shown their interest in several pathologies of children and adolescents. However, in this vulnerable population, they are not exempt from adverse effects. Hyperprolactinemia is a frequent and underestimated consequence of treatment with these drugs.

Risperidone has a marked tendency to elevate prolactin and induce the impact of hyperprolactinemia, comparable to haloperidol, and higher than most atypical antipsychotics. Reported prevalences range from 43.2% to over 64% [4].

Aripiprazole is more neutral, even decreasing prolactin levels. Several studies have affirmed this nature, hence its usefulness and effectiveness in the management of antipsychotic-induced hyperprolactinemia.

**Objectives:**

To highlight the importance of monitoring prolactinemia in children on antipsychotic drugs. evoke the different therapeutic alternatives for the management of this adverse effect. show the effectiveness of aripiprazole in the management of antipsychotic-induced hyperprolactinemia.

**Methods:**

We report the case of a 14-year-old girl, followed since the age of 5 for an intellectual development disorder, who was put on risperidone to manage her aggressiveness and insomnia. the appearance of mild hirsutism (Ferriman and Gallwey score = 15) with amenorrhea for 3 months. Thus, we decreased the dose of risperidone to 1 mg/d and requested a prolactinemia, which came back very high at 1637 mUI/l (N=63.6 - 305.28). The diagnosis of antipsychotic-induced hyperprolactinemia was retained after elimination of a prolactinoma and the patient was put on aripiprazole according to the modalities of the antipsychotic switch. We report the case of a 14-year-old girl, followed since the age of 5 for an intellectual development disorder, who was put on risperidone to manage her aggressiveness and insomnia.the appearance of mild hirsutism (Ferriman and Gallwey score = 15) with amenorrhea for 3 months. Thus, we decreased the dose of risperidone to 1 mg/d and requested a prolactinemia, which came back very high at 1637 mUI/l (N=63.6 - 305.28). The diagnosis of antipsychotic-induced hyperprolactinemia was retained after elimination of a prolactinoma and the patient was put on aripiprazole according to the modalities of the antipsychotic switch.

**Results:**

We observed a rapid decrease in serum prolactin as soon as 10 mg of aripiprazole was reached with a change from 1276 to 461 mIU/l after one month before its normalization the following month (237 mIU/l).

**Conclusions:**

The prescriber must therefore make a choice that is adjusted to the patient’s pathology, but also to the slightest sign of adverse effects. He will have to re-evaluate regularly the efficacy of the treatment and confront it with the possible adverse effects of the patient.

**Disclosure of Interest:**

None Declared

